# Introducing the TrypanoGEN biobank: A valuable resource for the elimination of human African trypanosomiasis

**DOI:** 10.1371/journal.pntd.0005438

**Published:** 2017-06-01

**Authors:** Hamidou Ilboudo, Harry Noyes, Julius Mulindwa, Magambo Phillip Kimuda, Mathurin Koffi, Justin Windingoudi Kaboré, Bernadin Ahouty, Dieudonné Mumba Ngoyi, Olivier Fataki, Gustave Simo, Elvis Ofon, John Enyaru, John Chisi, Kelita Kamoto, Martin Simuunza, Vincent P. Alibu, Veerle Lejon, Vincent Jamonneau, Annette Macleod, Mamadou Camara, Bruno Bucheton, Christiane Hertz-Fowler, Issa Sidibe, Enock Matovu, for the TrypanoGEN Research Group as members of The H3Africa Consortium

**Affiliations:** 1 Centre International de Recherche-Développement sur l’Elevage en zone Subhumide (CIRDES), Bobo-Dioulasso, Burkina Faso; 2 Centre for Genomic Research, University of Liverpool, Liverpool, United Kingdom; 3 College of Veterinary Medicine, Animal Resources and Bio-security, Makerere University, Kampala, Uganda; 4 Research Unit in Bioinformatics (RUBi), Department of Biochemistry and Microbiology, Rhodes University, Grahamstown, South Africa; 5 Université Jean Lorougnon Guédé (UJLoG), Daloa, Côte d’Ivoire; 6 Institut National de Recherche Biomedicale, Kinshasa, Democratic Republic of Congo; 7 Faculty of Science, University of Dschang, Dschang, Cameroon; 8 University of Malawi, College of Medicine, Department of Basic Medical Sciences, Blantyre, Malawi; 9 Department of Disease Control, School of Veterinary Medicine, University of Zambia, Lusaka, Zambia; 10 Institut de Recherche pour le Développement (IRD), Montpellier, France; 11 Institut Pierre Richet, Bouaké, Côte d’Ivoire; 12 Wellcome Trust Centre for Molecular Parasitology, University Place, Glasgow, United Kingdom; 13 Programme National de Lutte contre la Trypanosomose Humaine Africaine, Conakry, Guinea; Yale University, UNITED STATES

## Background

Human African trypanosomiasis (HAT), or sleeping sickness, is a disease caused by 2 subspecies of the protozoan parasite, *Trypanosoma brucei* (*T*. *b*.): *T*. *b*. *gambiense* and *T*. *b*. *rhodesiense*. *T*. *b*. *gambiense* causes the chronic form of sleeping sickness in West and Central Africa, and it is responsible for 98% of all reported cases [1]. *T*. *b*. *rhodesiense* causes an acute, rapidly progressive infection in Eastern and Southern Africa. Over the last decade, control measures have reduced HAT incidence to less than 3,000 reported cases in 2015, the lowest level in 75 years [2]. The target of the World Health Organization (WHO) is the elimination of the disease as a public health problem by 2020 and interruption of its transmission by 2030 [1].

HAT has been considered as an invariably fatal disease. However, recent studies indicate that this is not the case [3–5]. Infection by *T*. *b*. *gambiense* can result in a wide range of clinical outcomes in its human host [3–5]. This has been illustrated by the descriptions of self-cure in HAT patients refusing treatment in Ivory Coast [6] and the report of a patient who developed sleeping sickness in the United Kingdom 29 years after he last left an endemic area [7]. Furthermore, individuals with an elevated response to the card agglutination test for trypanosomiasis (CATT) and positive to the specific *T*.*b*. *gambiense* immune trypanolysis test, but who remain negative to available microscopic parasitological tests for more than 2 years, have been reported in West Africa and are believed to harbor latent infections [8,9]. These observations suggest that individuals infected with *T*. *b*. *gambiense* may exhibit variable degrees of susceptibility, and some may be able to control the infection over long periods of time, as has also been demonstrated for African Animal Trypanosomiasis (AAT), caused by *T*. *congolense* [10,11].

The TrypanoGEN network is composed of researchers from Burkina Faso, Cameroon, Côte d’Ivoire, Guinea, Democratic Republic of Congo (DRC), Malawi, Uganda, Zambia, France, the UK, and Belgium and is aimed at understanding the genetic basis of human susceptibility to trypanosomiasis. The TrypanoGEN network is a member of the Human Heredity and Health in Africa (H3Africa www.h3africa.org) Consortium, which is an initiative funded and supported by the Wellcome Trust (UK) and the National Institutes of Health (United States). Members of the H3Africa Consortium are undertaking genome-wide association studies (GWAS) on a wide range of communicable and noncommunicable diseases. H3Africa members are committed to making all specimens and data available to other researchers [12].

The objectives of the TrypanoGEN network are to identify the human genetic determinants of disease susceptibility/resistance in different African populations and to determine if these are shared for the 2 diseases caused by the different trypanosome subspecies. To address these scientific questions, the TrypanoGEN project has created a biobank of specimens with standardized parasitological and clinical data. HAT is a disease of rural Africa, often in areas with limited health resources and impoverished populations. The diagnosis of trypanosomiasis, and particularly of subclinical cases, requires multiple tests in both the field and the laboratory. Large specimen collections from cases and individuals with latent infections require large surveys in areas that are remote from research laboratories. Both these factors make it hard to obtain large numbers of well-phenotyped specimens. To date, all the genetic association studies on the susceptibility in HAT have been performed on limited numbers of specimens sampled in single countries [13–16]. Thus, the TrypanoGEN biobank is an important resource to enable the first GWAS of HAT using a large and well-characterized specimen collection originating from different populations. A GWAS will make it possible to identify genetic determinants of susceptibility/resistance, validate hypotheses developed in the laboratory, and facilitate the development or evaluation of new diagnostic tests.

In this article, we describe the TrypanoGEN network biobank for human African trypanosomiasis, where all specimens used in our research are archived and made available to other researchers. Another biobank of specimens from HAT patients and endemic controls has been established previously by the WHO, but this is exclusively for the development of HAT diagnostic tests, and specimens are not available for genetic research [17].

## What are the characteristics and requirements of the HAT biobank?

### What ethical approval was obtained?

Ethical approval was provided by the national ethics councils of each of the TrypanoGEN participating countries: Cameroon (2013/364/L/CNERSH/SP), Democratic Republic of Congo (No 1/2013), Guinea (1-22/04/2013), Cote d’Ivoîre (2014/No 38/MSLS/CNER-dkn), Malawi (1213), Uganda (HS 1344), and Zambia (011-09-13). Information (in English or French and several local languages) was provided to the participants through community engagement with potential participants and community leaders at every field visit. This information included the scientific objectives of the TrypanoGEN network. The participants were guided through the consent forms, and written consent was obtained to collect biological specimens. This consent represents a broad consent that allows for genomic investigations as part of the TrypanoGEN network. Genetic studies on HAT by other researchers are allowed but require approval from the institutional review boards of the country that supplied the specimens.

### From whom were specimens collected?

**Fig 1:**
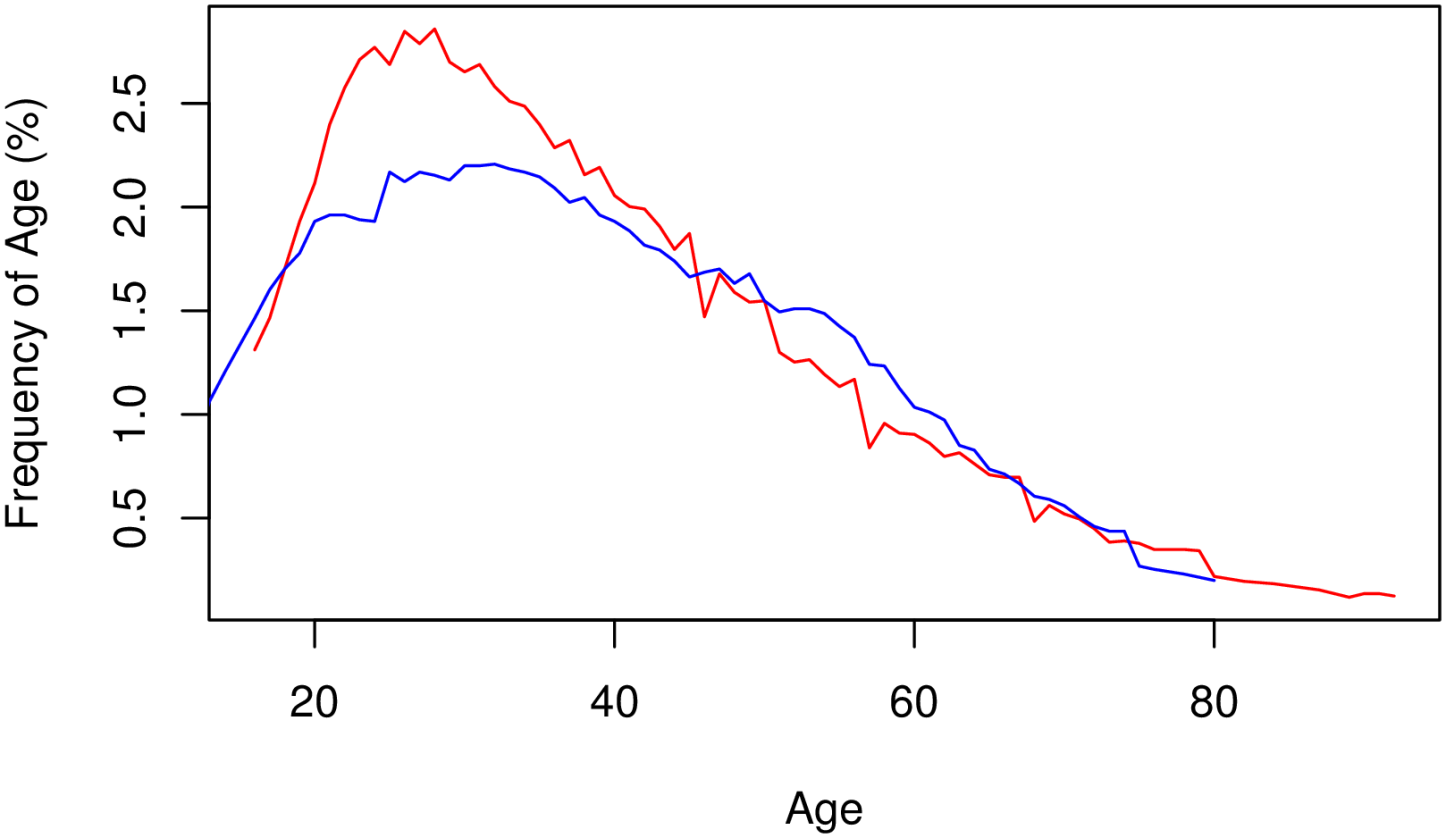
Age frequencies of case and control participants. Many participants did not know their age, and approximate ages were assigned to many subjects, leading to large spikes of numbers at 30, 40, 50, and 60 years, with minor spikes at 5-year intervals. These spikes were smoothed using a moving-window average over 10 years. The mean ages of *Trypanosoma brucei gambiense* and *T*. *b*. *rhodesiense* case and control participants were 36 and 42, respectively. The age distributions were similar except that the *T*. *b*. *rhodesiense* subjects’ ages were shifted to the right.

Study participants were classified into 3 groups depending on the serological and parasitological results:

- Cases, defined as individuals with trypanosomes detected in either lymph node aspirate, blood, or cerebrospinal fluid (CSF) by microscopy, after concentrating parasites, if necessary [18–21].
- Individuals with latent infections, with positive CATT [22] and trypanolysis test results [23], in whom no trypanosomes were detected by microscopy for at least 2 years.
- Controls, defined as individuals living in an endemic area who were CATT and trypanolysis negative, with no signs or symptoms suggestive of HAT and without evidence of previous HAT infection. The control participants had similar age and gender distributions as the case participants (Fig 1).

In total, the TrypanoGEN biobank currently includes 1,345 cases, 179 individuals with latent infections, and 1,777 controls (Table 1).

**Table 1:** Number of individuals by country with test results that meet the criteria for specimen classification.

Country	Cases	Latent Infections	Controls
***Trypanosoma brucei gambiense***			
Guinea	346	89	193
Ivory Coast	123	18	271
Democratic Republic of the Congo	213	72	282
Cameroon	56	-	160
Uganda	157	-	227
***T*. *b*. *rhodesiense***			
Uganda	245	-	372
Malawi	162	-	200
Zambia	39	-	62
Total	1,345	179	1,777

### How were specimens collected?

Specimens for the biobank were generally collected during field surveys in collaboration with national control programs in 3 different ways:

- Previously archived specimens: existing collections of previously archived specimens were centralized into their respective network node. For previously archived specimens, all individuals were revisited to obtain consent.
- Retrospectively collected specimens: study sites were revisited to consent and resample previously diagnosed and treated patients and individuals with latent infections.
- Prospectively collected specimens: HAT case patients before treatment and individuals with latent infections were identified as part of active surveillance surveys performed by the spoke teams in collaboration with their respective National Control Programs (NCP).

Whole blood, plasma, and buffy coat (BC) were collected from each study participant following good clinical practice principles. The specimens were frozen in the field at −20°C before final storage at −80°C.

Epidemiological and laboratory data were recorded and linked to biological specimens (Table 2). Plasma was used to perform the trypanolysis test. Blood or BC was used for DNA extractions using the Whole Blood Midikit from Qiagen. DNA was quantified by fluorometric Qubit assay (Invitrogen) or Nanodrop spectrophotometer.

**Table 2:** Metadata associated with each specimen in the database.

Epidemiological Data	Laboratory Data
ID	Trypanolysis result
Country	Parasitological result
Focus	
Village	
Sex	
Age	
Language	

### Where were specimens collected and stored?

The biobank stores plasma and DNA from blood in 3 hubs that are responsible for specimen management and associated data, including reception, control, processing, storage, and subsequent distribution to end users. Shipment from intermediate national storage to the central hubs was by express courier on dry ice.

**Fig 2:**
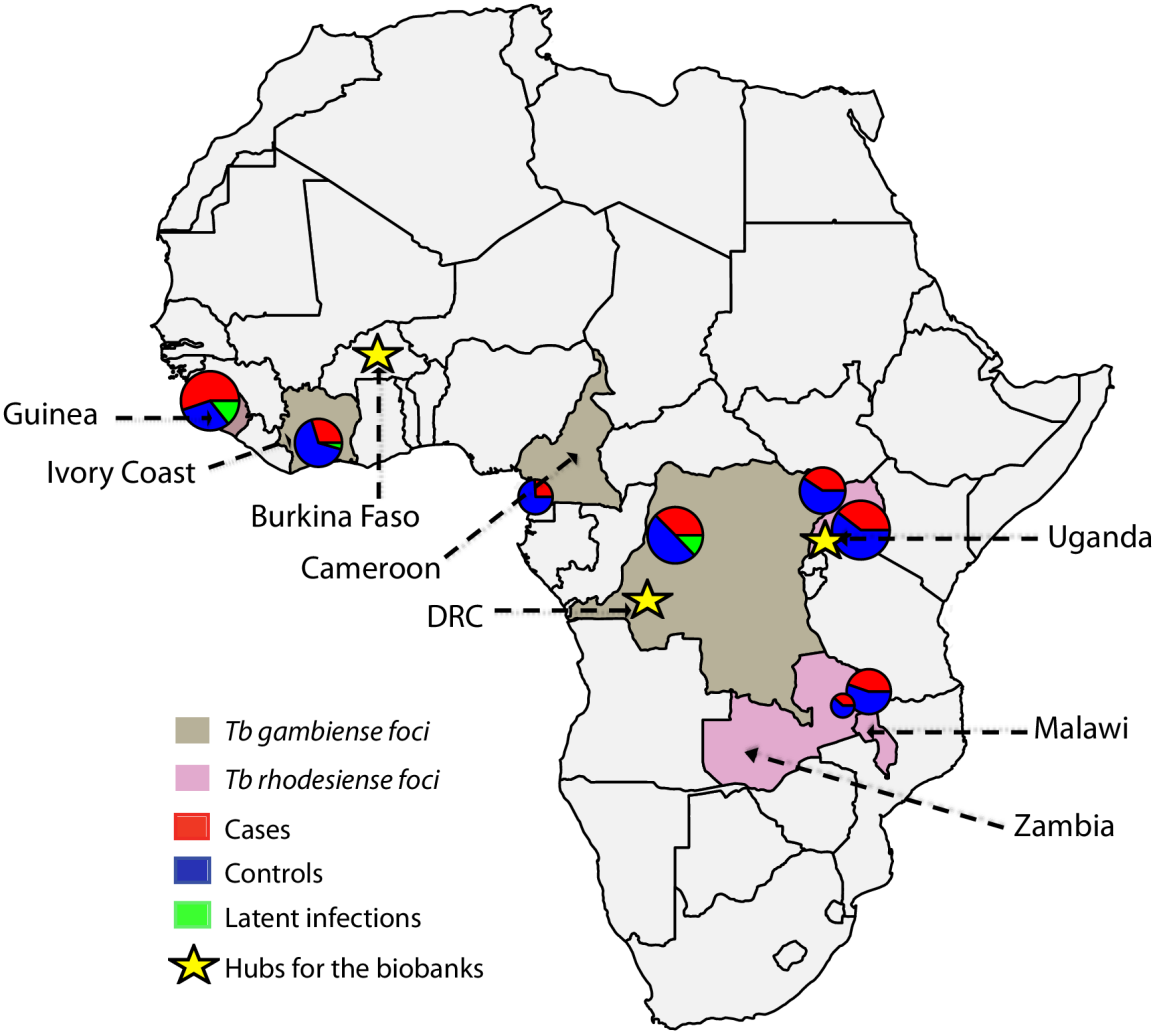
Map showing countries from which samples were collected and the proportion of each type of subject in the collection. Stars indicate locations of biobank hubs.

A map of the TrypanoGEN network is given in Fig 2. Specimens are held at the following locations:

- The College of Veterinary Medicine, Animal Resources & Bio-security (COVAB), Makerere University, Kampala, Uganda. This location preserves specimens from the *T*. *b*. *gambiense*–endemic districts of northwestern Uganda and the *T*. *b*. *rhodesiense*–endemic districts of eastern Uganda as well as from the Rumphi and Nkhotakota foci in Malawi and multiple districts of Zambia.
- Centre International de Recherche-Développement sur l’Elevage en zone Subhumide (CIRDES), Burkina Faso, which holds specimens from Forécariah, Dubréka, and Boffa foci collected by the National Control Programme of Guinea and from Bonon and Sinfra foci in Côte d’Ivoire, collected by the Université Jean Lorougnon Guédé, Daloa, Côte d’Ivoire.
- Institut National de Recherche Biomedicale (INRB), Kinshasa, DRC, holds specimens from Bandundu, DRC, and from the Campo and Bipindi foci in Cameroon, collected by the Faculty of Science, University of Dschang, Cameroon.

### What metadata are associated with specimens?

Metadata associated with each specimen are shown in Table 2. Phenotyping was focused solely on HAT; no screening was done for other infections. Whole genome sequencing to 10X coverage has been performed on 298 DNA specimens from 6 countries. Case patients, individuals with latent infections, and control individuals will be genotyped using the H3Africa 2.5 million single nucleotide polymorphism (SNP) chip genotyping assay [12]. The sequence data and SNP chip data will be associated with the subjects and made freely available under the standard H3Africa data release policy 1 year after data collection is completed. Guidelines and information on the policies for specimens and data sharing are accessible through the H3Africa website (www.h3africa.org) [12].

### How can users access the TrypanoGEN biobank?

The specimens are accessible to the members of the network for further research on HAT after review of applications by the sample access committee and are subject to a material transfer agreement. Interested researchers from outside the TrypanoGEN network should request access to the biobank through the principle investigators (PIs) of the 3 hubs, who will refer requests to the TrypanoGEN sample access committee so that they can find if there are specimens available that meet the needs. Contact details of the PIs are available from the project website: www.trypanogen.net.

The TrypanoGEN biobank database is web accessible and allows the user to browse and filter TrypanoGEN subjects to identify sets of subjects that might be appropriate for a particular study. Access to the database is available on request to the PIs of the hubs. Access will be provided to researchers to evaluate the utility of the specimens for their purpose and make specific requests for specimen sets.

**Key learning points**

- The rapidly declining cost of genetic assays means that specimen collection is now the most expensive, laborious, and time-consuming part of genetic research. The biobank will share this valuable resource to facilitate studies that would not otherwise be possible.
- The H3Africa Consortium members are setting up biobanks for specimens associated with a wide range of conditions. This is an opportune moment to set up collaborations with research questions cutting across regions.
- Experimental reproducibility is a hot current topic [24]. The availability of a large standard set of specimens from multiple populations will allow researchers to undertake well-powered experiments that can reveal both population-specific and Africa-wide associations with trypanosomiasis that can be tested for reproducibility by other researchers.
